# Proliferation and osteogenic activity of fibroblasts induced with fibronectin

**DOI:** 10.1590/1414-431X20176272

**Published:** 2017-08-17

**Authors:** W.-H. Zhang, X.-L. Li, Y. Guo, Y. Zhang

**Affiliations:** Tianjin Hospital, Tianjin, China

**Keywords:** Fibroblast, Fibronectin, Proliferation, Osteogenesis

## Abstract

The aim of this study was to determine the proliferation and osteogenic activity of fibroblasts induced with fibronectin and their possible dose-dependent relationship. The fibroblasts obtained by tissue explants adherent method were induced with fibronectin at different concentrations of 0, 10, 20, 40, 60, and 80 μg/mL for 14 days. The ^3^H-thymidine and ^3^H-proline incorporation test was used to evaluate the synthesis of DNA and collagen by fibroblasts, respectively. The mineralized nodules and osteocalcin secretion, as vital osteogenic indicators, were detected with tetracycline labeling and ^125^I-labeled competitive immunoassay, respectively. Fibronectin significantly increased the synthesis of DNA and collagen by fibroblasts, especially at the concentration of 40 μg/mL (P<0.05). The increased secretion of osteocalcin in the supernatant was also statistically significant at the concentration of 40 μg/mL (P<0.05). The mineralized nodules with trabecula-like structure derived from induced fibroblasts were positive for tetracycline labeling. The granulation tissue-derived fibroblasts induced with fibronectin exhibited increased proliferative, functional and osteogenic potential. Fibroblasts are considered a possible *in situ* stem cell in tissue engineering.

## Introduction

The infectious soft tissue and bone defects induced by trauma is often followed by bone non-union and osteolysis and has been a critical problem to be solved by orthopedic surgeons. These patients experience poor limb function and decreased life quality. Most orthopedic surgeons make great efforts to treat infectious soft tissue and bone defects using Western medicine. The first step is to reconstruct the soft tissue coverage of the defect area with flaps rich in blood supply, aiming to control infection with the assistance of efficient antibiotics. Secondarily, bone graft and rigid internal fixation are used to treat bone nonunion (1,2). However, the results are not certain.

In the field of traditional Chinese medicine, the medicinal herbal extract named ShengJi Ointment (Tianjin Hospital, Tianjin, China) is applied to the surface of infectious soft tissue and bone defect. With time, granulation tissue exhibits growth and re-epithelization, and gradually covers the soft tissue defect. More interestingly, the island-shaped mineral-containing granulation tissue can be seen in the bone defect area, which gradually fuses and links the fragments of the fracture. It simultaneously repairs the infectious soft tissue and the bone defect. In the procedure, the secretion from the wound area, derived from the interaction of the local tissue and ShengJi Ointment, is fester-like and thought to play an important part in the healing. What could be responsible for the mineralization of the granulation tissue in the bone defect area? It is known that the main cell component of the granulation tissue is the fibroblast and the main biomolecule of the fester-like secretion is fibronectin (3). Therefore, our experiment was designed to determine whether the granulation tissue-derived fibroblasts show osteogenic activity when induced with fibronectin, and to determine their possible dose-dependent relationship in the proliferation of fibroblasts and osteogenesis.

## Material and Methods

### Animal experiments and fibroblasts isolation

A circular full-thickness skin defect, with a diameter of 2 cm, was made on the back of two healthy adult rabbits (New Zealand rabbits, Institute of Laboratory Animal Sciences, CAMS & PUMC) under aseptic condition. After 3 to 5 days, granulation tissue appeared in the circular area of the skin defect. We acquired the granulation tissue and cut it into 1×1 mm pieces. The fragments of granulation tissue were washed three times with Hanks solution (Invitrogen, USA), plated in DMEM (Invitrogen) supplemented with 10% fetal bovine serum (Gibco, USA) and 1% mycillin (Invitrogen), in a 50-mL culture bottle, and incubated at 37°C with 5% CO_2_. Adherent fibroblasts were harvested using 0.25% trypsin-EDTA (Invitrogen), and re-plated at 1×10^5^/mL in the medium described above.

All animal use protocols were reviewed and approved by the Animal Management Committee of Tianjin City (China).

### Fibroblast culture

Passage 3 fibroblasts were plated at 2×10^4^ cells per well in a 24-well plate and incubated at 37°C with 5% CO_2_. After the cells adhered overnight, fibronectin (Sigma, USA) was added to the medium at the following concentrations: 0 (control group), 10, 20, 40, 60, and 80 μg/mL. Cells were incubated at 37°C for 14 days, with three wells for each group. The medium containing fibronectin was regarded as a conditioned culture. The medium of each group was replaced every 3 days.

### Cell morphology

The changes in cell morphology, cell structure and mineralized nodule formation were visualized every day with hematoxylin and eosin (HE) staining using an inverted phase contrast microscope (Olympus, Japan).

### Mineralized nodule labeling with tetracycline

Tetracycline staining was used to demonstrate mineralization. After 14-day culture, PBS solution (Invitrogen) containing tetracycline (Sigma) at 50 μg/mL was added to each well with incubation at 37°C for 1 h. The cells were incubated for another 1 h with the above medium, washed twice with Hanks solution and fixed with 80% cold alcohol (Invitrogen). Cell mineralization was visualized with a fluorescence microscope (Olympus).

### Labeling with ^3^H-thymidine

The proliferative capacity of fibroblasts was evaluated with the amount of DNA synthesis labeled with ^3^H-thymidine (^3^H-TdR; China Institute of Atomic Energy, China), namely the ^3^H-TdR incorporation test. After fibroblasts were cultured for 7 and 14 days, ^3^H-TdR at 9.25 kBq/mL (1 Ci=3.7×10^10^Bq) was added to the medium and incubated at 37°C for 24 h. The cells were collected, washed, dried at 80°C and detected by a liquid scintillation counter (Perkin Elmer, USA) with the result reported as count per minute (cpm).

### Labeling with ^3^H-proline

The amount of collagen synthesis labeled with ^3^H-proline (China Institute of Atomic Energy) was assessed by a liquid scintillation counter (Perkin Elmer) with the result reported as cpm. The method was the same as that performed for ^3^H-TdR detection.

### Osteocalcin assessment

Osteocalcin is regarded as an important indicator of osteogenesis. The supernatant was collected to assess the amount of secreted osteocalcin with ^125^I-labeled competitive immunoassay, after fibroblasts were cultured for 4, 7, and 14 days. The procedures were performed according to the description of the osteocalcin kit (Invitrogen). The unit of measurement was μg/L.

### Statistical analysis

Data are reported as means±SD for each group. Comparisons between groups were performed by one-way analysis of variance (ANOVA) with version 12.0 of Statistical Package of Social Sciences software (SPSS Inc., USA). P<0.05 was considered to be statistically significant.

## Results

### Cell morphology

**Figure 1.**
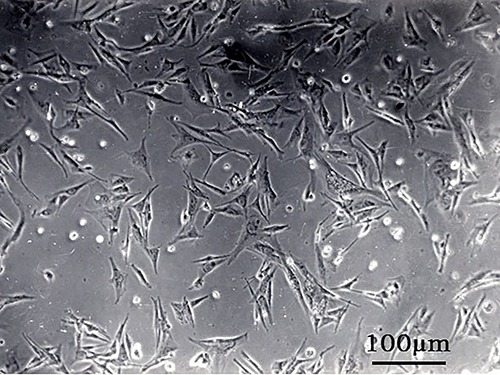
After seeding for 24 h, passage 3 fibroblasts adhered and stretched. Phase contrast microscope, ×100.

**Figure 2.**
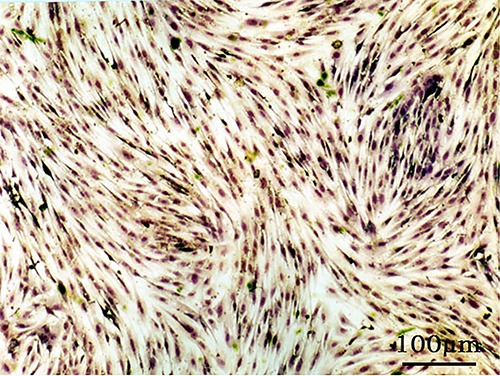
The control group fibroblasts reached confluence and displayed a fingerprint-shaped order at 5 days. HE staining, ×100.

Passage 3 fibroblasts began to adhere after seeding for 2 h. After 24 h, all fibroblasts adhered and stretched (Figure 1). The control group fibroblasts looked spindle-shaped, reached 100% fusion rate after 3 days and displayed a fingerprint-shaped order after 5 days of incubation (Figure 2). As days passed, fibroblasts proliferated slowly and dropped sporadically. When cultured for 14 days, fibroblasts had little mineralized nodules.

**Figure 3.**
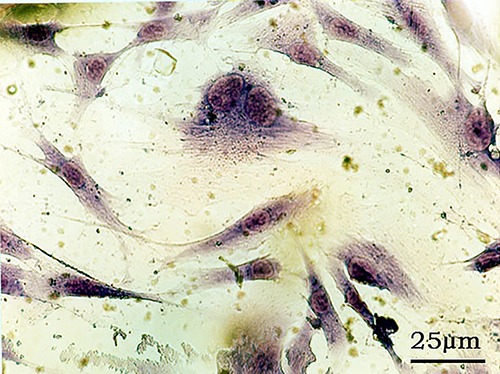
Fibroblasts induced with 40 μg/mL fibronectin had decentered nucleus and distinct particles in cytoplasm at 5 days. HE staining, ×200.

**Figure 4.**
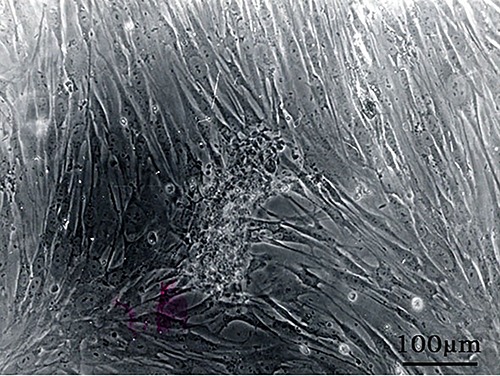
Nebulous extracellular secretions covered fibroblasts induced with 40 μg/mL fibronectin at 7 days. Phase contrast microscope, ×100.

**Figure 5.**
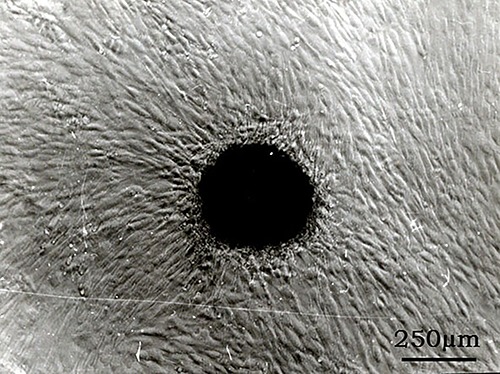
Dark tight nodules formed at 14 days in fibroblasts induced with 40 μg/mL fibronectin. Phase contrast microscope, ×100.

The fibroblasts in groups conditioned with fibronectin showed stronger proliferation compared with those of the control group. The fibroblasts looked triangle-shaped and exhibited obvious karyoschisis in the nucleolus after 3 days of culture. After culture for 5 days, fibroblasts had a decentered nucleus and distinct particles in cytoplasm (Figure 3). After 7 days, there were nebulous extracellular secretions covering cells (Figure 4). As days passed, nebulous secretions gathered and formed dark nodules (Figure 5). The dark nodules gradually fused and formed a trabecula-like structure.

### Mineralized nodule labeling with tetracycline

**Figure 6.**
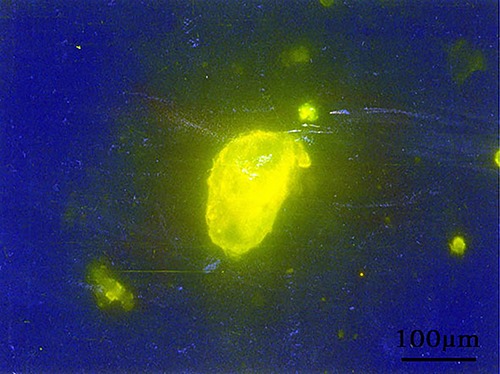
Tetracycline labeling for fibroblasts induced with 40 μg/mL fibronectin showing golden nodules, which had a trabecula-like structure. Fluorescence microscope, ×100.

The visualized dark nodules were positive for the tetracycline staining, indicating mineralization. Compared with the control group, there were more and larger golden nodules containing a trabecula-like structure in the conditioned groups, especially in the group with a fibronectin concentration of 40 μg/mL (Figure 6).

### Labeling with (^3^H-TdR)

**Figure 7.**
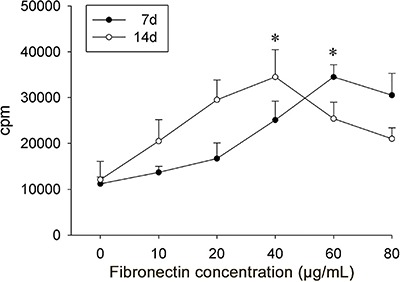
^3^H-TdR incorporation test indicating that fibronectin regulated the DNA synthesis of fibroblasts. The 60 μg/mL fibronectin concentration at 7 days and the 40 μg/mL fibronectin at 14 days had significantly higher DNA synthesis compared to the other groups (*P<0.05, ANOVA). Data are reported as means±SD. cpm: counts per minute.

The results of the ^3^H-TdR incorporation test indicated the amount of the fibroblast DNA synthesis. Fibronectin regulated the DNA synthesis of fibroblasts. The group with a fibronectin concentration of 60 μg/mL at 7 days and the group with a fibronectin concentration of 40 μg/mL at 14 days had significantly higher DNA synthesis (P<0.05) compared with the other groups (Figure 7).

### Labeling with ^3^H-proline

**Figure 8.**
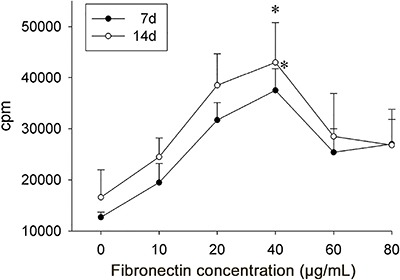
^3^H-proline incorporation test showing that fibronectin up-regulated the collagen synthesis of fibroblasts. Collagen synthesis in the 40 μg/mL fibronectin group was significantly higher at 7 and 14 days than the other groups (*P<0.05, ANOVA). Data are reported as means±SD. cpm: counts per minute.

Fibronectin up-regulated the collagen synthesis of fibroblasts. The group with fibronectin concentration of 40 μg/mL peaked at 7 and 14 days (P<0.05), the highest among all the groups (Figure 8).

### Osteocalcin assessment

**Figure 9.**
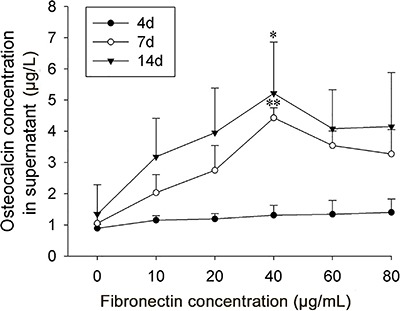
Osteocalcin concentration increased with culture time in all groups. The 40 μg/mL fibronectin was statistically significant at 7 (**P<0.01) and 14 days (*P<0.05) compared to the other groups (ANOVA). Data are reported as means±SD.

The increasing osteocalcin amount showed the osteogenesis capacity of fibroblasts induced by fibronectin. There was no statistical significance among groups at day 4 (P>0.05). The group of 40 μg/mL fibronectin was statistically significant at day 7 (P<0.01) and day 14 (P<0.05), compared with the other groups (Figure 9).

## Discussion

In this study, fibroblasts were obtained from the granulation tissue by tissue explants adherent method. Fibronectin in different concentrations was used to induce fibroblasts, and the ability of proliferation and osteogenesis was evaluated. The ^3^H-TdR and ^3^H-proline incorporation test exhibited the high proliferative and functional capacity of fibroblasts, respectively. Fibronectin at 40 μg/mL dramatically up-regulated the proliferation and collagen synthesis of fibroblasts. The mineralized nodules and osteocalcin levels were vital osteogenic indicators. With the induction of fibronectin, fibroblasts looked triangle-shaped, proliferated, gathered with nebulous extracellular secretions and formed dark nodules ultimately. The nodules were positive for tetracycline-labeling and identified as mineralized nodules. Combined with the significantly increased osteocalcin levels, fibroblasts showed a definite osteogenic capacity when induced with fibronectin, especially at the concentration of 40 μg/mL. In summary, fibroblasts induced with fibronectin showed a high potential of proliferation, function and osteogenesis.

Some reviews have reported that fibroblasts derived from the adventitia (4), cardiac (5,6), burn scar (7) and normal skin (8) could be induced to myofibroblasts, which express α-smooth muscle actin. Periodontal fibroblasts were considered to have the potential of osteoblastic differentiation with an osteogenic medium containing 50 mg/mL of ascorbic acid-2-phosphate and 10 mM of β-glycerophosphate (9). Periodontal fibroblasts strongly expressed osteogenic genes such as ALP, Runx2, osterix and osteocalcin and formed mineralized nodules (10). The human dermal fibroblasts cultured on macroporous gelatin microcarriers encapsulated in platelet-rich plasma into three-dimensional constructs were successfully differentiated towards chondrogenic and osteogenic phenotypes using specific induction media (11). In 2005, Rieske et al. (12) reported that fibroblasts had some of the characteristics of stem cells, and could be induced to become neuroectodermal cells and perhaps even mature neurons. Moreover, the human vascular adventitial fibroblasts from pulmonary arteries, were cultured in appropriate media and showed osteogenic, adipogenic and myogenic differentiation (13).

Based on the above literature and our study on fibroblasts, we present the theory that fibroblasts have a potential of *in situ* pleuripotent stem cell. Under specific conditions, fibroblasts could exhibit pleuripotent differentiation in accordance with inductive factors. Mesenchymal stem cells (MSCs) are considered to be a promising source of stem cells in regenerative medicine. There has been evidence that MSCs could be induced to differentiate into fibroblasts with conditioned induction media (14). Perhaps fibroblasts are a kind of intermediate cell in the differentiation system from mesenchymal stem/progenitor cells to terminally differentiated cells, not strictly confined to differentiated fibrocytes.

The induced pluripotent stem (iPS) cells have also been extensively studied. Fully reprogrammed iPS cells display numerous properties similar to those of embryonic stem cells and exhibit the capacity of self-renewal and differentiation into three germ layers (15). Up to now, there are few documents that suggest the use of MSCs or iPS cells to treat infectious soft tissue and bone defects. A series of undergoing experiments at the molecular and cellular levels are rooted in clinical observations on the subject. Our experiment demonstrated the fibroblast capacity of proliferation and osteogenesis with the induction of fibronectins. More importantly, the results supported the clinical observations that the granulation tissue, whose main cellular component is the fibroblast, could treat infectious soft tissue and bone defects.

Why can fibroblasts survive in an infectious environment filled with virulent bacteria, such as *staphylococcus aureus* or/and *pseudomonas aeruginosa*, and display strong proliferation and osteogenesis? Although not tested in this study, a key factor could be the employment of the ShengJi Ointment, a Chinese herbal preparation. Our preliminary work reported that the ShengJi Ointment and its fester-like secretions effectively enhanced capillary permeability of the local soft tissue and activated a monocyte-macrophage system that could efficiently secret various bioactive molecules, including fibronectins, not aiming at killing bacteria (16–18). Thus, we regarded ShengJi as an efficient biological agent, due to its immune modulation function. Although fibronectins play a vital role in adhesion, migration, chemotaxis and opsonization (19), many factors contribute to the local environment in a synergistic way. The employment of the Chinese herbal preparation as a promising biological agent and inductive factor is a novel strategy to induce the pluripotent stem cells in tissue engineering.

In conclusion, our experiment demonstrated that granulation tissue-derived fibroblasts induced with fibronectin exhibited increased proliferative, functional and osteogenic potential. Fibroblasts are considered a possible *in situ* stem cell in tissue engineering.
